# FuncTree: Functional Analysis and Visualization for Large-Scale Omics Data

**DOI:** 10.1371/journal.pone.0126967

**Published:** 2015-05-14

**Authors:** Takeru Uchiyama, Mitsuru Irie, Hiroshi Mori, Ken Kurokawa, Takuji Yamada

**Affiliations:** 1 Department of Biological Information, Tokyo Institute of Technology, Tokyo, Japan; 2 Earth-Life Science Institute, Tokyo Institute of Technology, Tokyo, Japan; Georgia Institute of Technology, UNITED STATES

## Abstract

Exponential growth of high-throughput data and the increasing complexity of omics information have been making processing and interpreting biological data an extremely difficult and daunting task. Here we developed FuncTree (http://bioviz.tokyo/functree), a web-based application for analyzing and visualizing large-scale omics data, including but not limited to genomic, metagenomic, and transcriptomic data. FuncTree allows user to map their omics data onto the “Functional Tree map”, a predefined circular dendrogram, which represents the hierarchical relationship of all known biological functions defined in the KEGG database. This novel visualization method allows user to overview the broad functionality of their data, thus allowing a more accurate and comprehensive understanding of the omics information. FuncTree provides extensive customization and calculation methods to not only allow user to directly map their omics data to identify the functionality of their data, but also to compute statistically enriched functions by comparing it to other predefined omics data. We have validated FuncTree’s analysis and visualization capability by mapping pan-genomic data of three different types of bacterial genera, metagenomic data of the human gut, and transcriptomic data of two different types of human cell expression. All three mapping strongly confirms FuncTree’s capability to analyze and visually represent key functional feature of the omics data. We believe that FuncTree’s capability to conduct various functional calculations and visualizing the result into a holistic overview of biological function, would make it an integral analysis/visualization tool for extensive omics base research.

## Introduction

Recent advancement in high throughput DNA sequencing technology has lead to an exponential growth of omics information. Additionally, the recent development of methodology such as metagenomic analysis, where biologists have to deal with the genetic information of multiple organisms and various meta-data, instead of a single cell or an organism, has lead to the increase complexity of omics information. As a result, in contrast to the advancement of high throughput sequencing technology, comprehending omics data in order to develop further biological insights is becoming an increasingly difficult and daunting task.

A key solution to this problem is data visualization. Transforming complex data in to visual expression that is comprehensible to human cognition is essential for biologist to discover key features and characteristics hidden in the data. Consequently, developing new visualization methods that projects vast and complex omics information in to an intuitively comprehensible visual expression is becoming increasingly important.

One field that we want to focus in this research is the field of functional genomics. Conventional methods to visually express the biological functionality of an omics data, is to draw a pie chart, bar graph, or to map the data onto a pre-defined molecular network map, such as chemical pathways[1,2]. The problem with these conventional methods is that visualization is only limited to functions under a particular functional category, which are often times selected arbitrarily. As a consequence, it is difficult to overview the broad functional potential of a particular omics data using conventional visualization techniques. This is particularly problematic when you consider the fact that, different functional category may show different unique features depending on the data, and we still do not have enough understanding to tell us, which functional category to focus on, for a given type of data. In order to address this problem, we have developed a new data analysis and visualization tool, FuncTree, which aims to intuitively and holistically visualize the functional potential of large-scale omics data.

## FuncTree overview

FuncTree is an open-access online web application for analyzing and visualizing the functional potential of large-scale omics information, such as genomic, metagenomic, and transcriptomic data. FuncTree achieves this by mapping the omics data onto a "Functional Tree", a pre-defined hierarchical tree map, which visually represents the hierarchically classification of biological functions defined in the KEGG database [3].

KEGG classifies biological functions into mainly three functional layers. The first is the KEGG Orthology (KO), which consists of manually defined ortholog groups for all proteins and functional RNAs. Most KOs are associated with a particular enzyme. The second layer is the KEGG Module, which represents tight functional unit or chemical reaction. KOs that catalyzes the chemical reaction defined by a particular module is grouped together and assigned to it. The third layer is the KEGG Pathway, which is a collection of modules that drives similar molecular interaction.

**Fig 1:**
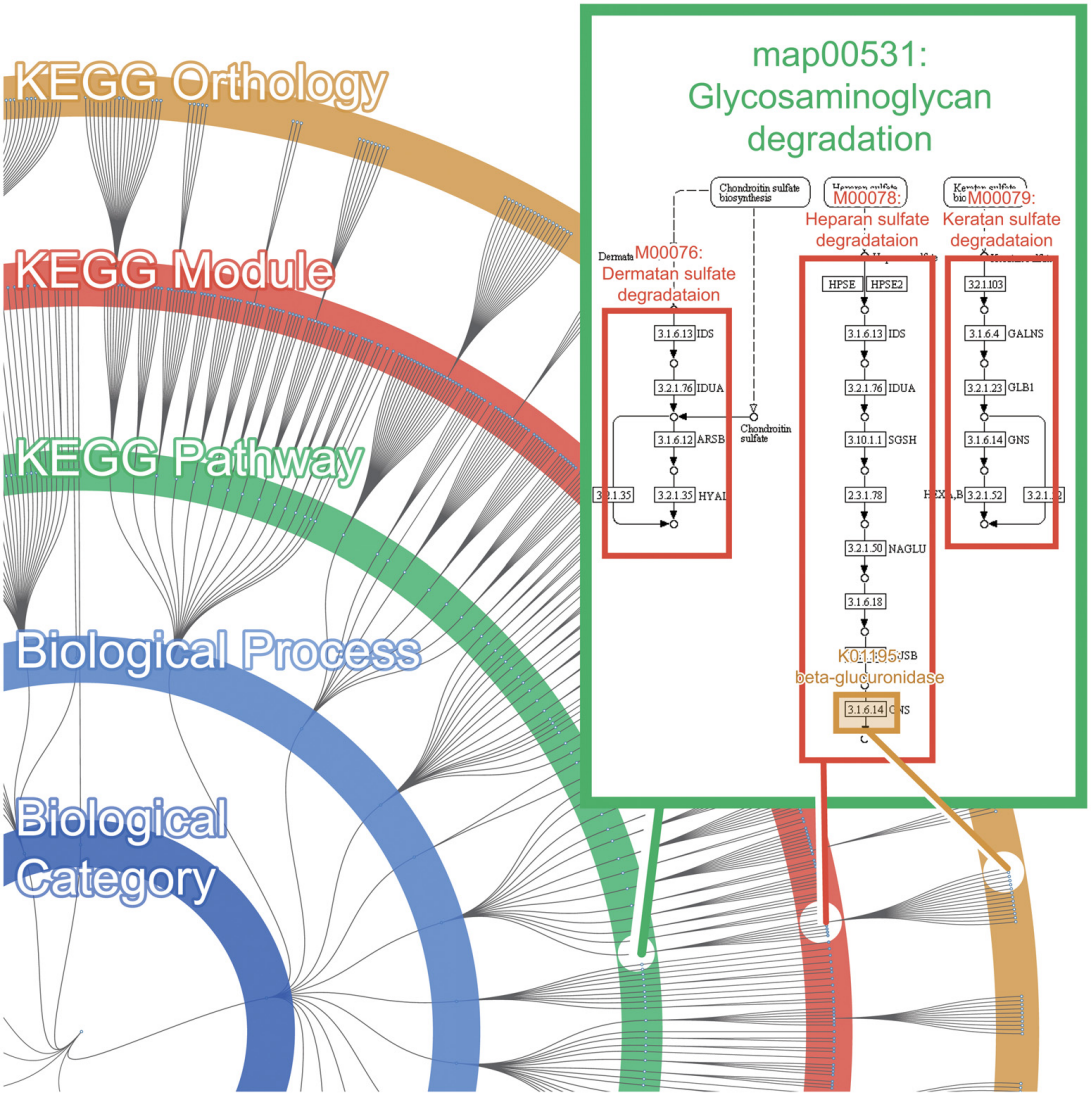
Overview of the Functional Tree map. The Functional Tree is a circular dendrogram that represents the hierarchical relationship of known biological functions defined in the KEGG database. Each layer of the tree corresponds to each functional category, “Biological Category”, “Biological Process”, “KEGG Pathway”, “KEGG Module”, and the outer most layer corresponds to “KEGG Orthology”. Each layer contains multiple nodes that correspond to the biological functions that are assigned to that functional category.

The Functional Tree is comprised of 28,505 KOs, which are assigned to all 676 modules, and all 365 pathways. The 365 pathways and 676 modules are part of the existing KEGG BRITE database, however the hierarchical relationship between module and pathway are not explicitly shown in the BRITE hierarchy. Since the hierarchical relationship between module and pathway can be inferred by looking at the inclusion relationship of pathway-KO and module-KO, we have used this information to reconstruct the BRITE hierarchy, so that it incorporates the information of modules. Additionally the BRITE hierarchy does not contain the information regarding KOs that are unassigned to pathways. Therefore, we have added 7,818 KOs (pre-existing in KEGG database) that were unassigned to pathways. For the 7,818 KOs that were not assigned to any modules or pathways, the tree hides these visually, under "Undefined Biological Category". However FuncTree does includes these hidden KOs for calculation and normalization purposes. FuncTree represents all 37,364 biological functions as a node within the Functional Tree. The edge connecting each node represents the hierarchical relationship between the different functions (Fig 1).

By incorporating this new visualization method, FuncTree is able to visualize the functionality of omics data across different functional layers (KO, Module, Pathway), which allows users to overview the broad functional potential of their data. Additionally, by mapping omics data onto the Functional Tree, user can accurately identify the expression of overlapping functions, such as module and KO that are assigned to multiple different pathways. Conventionally, even if the expression of certain modules and KOs were identified, it was difficult to infer to which pathway those module/KO were assigned to, thus making it difficult to attach any broader biological meaning to the expression of that module/KO. By mapping the omics data onto the Functional Tree, user can view how many sub-functions (e.g. module, KO) are expressed under each biological function (e.g. pathway, module), which allows users to accurately identify, for example, enriched pathway that has overlapping KOs with other pathways.

FuncTree also provides basic statistical analysis features, which enables user to conduct enrichment analysis and seamlessly view the result using FuncTree alone.

### UI and basic functionality

FuncTree provides basic panning and zooming control to navigate through the tree map. Mouse-over on a node to open a tooltip showing further detailed information about the function. Clicking the node will expand or collapse the children nodes assigned under it. This allows users to manually customize the default tree map to suit their own data type. FuncTree also allows user to customize the tree map to center on a particular node. This allows user to focus on a particular functional category rather than viewing the entire Functional Tree. FuncTree also outputs the result in table format with basic sorting and searching mechanism. We believe that this conventional analytical representation of omics data will be extremely useful when combined with Functional Tree's more intuitive and graphical representation of omics data’s functionality.

Omics data may be uploaded via plain text query or file upload. FuncTree expects a list of node (biological function) name and an associated value. This value can represent various variables, such as the intensity of gene expression or a p-value showing the statistical significance of the over-represented genes. FuncTree will draw a colored circle on the node, corresponding the input node name, with a radius, corresponding to the input value.

FuncTree provides extensive visual customization options to achieve optimal visualization for various data. Additionally to "value" or radius size, user can also define "color" and "opacity" for each nodes. Optionally user can select different normalization methods to determine how each value will be graphically represented. For example user can decide whether the node value would be represented by the circle's radius length or area size. For further detail information about input parameters and example data, and a full list of customization option, refer to FuncTree's online help page (http://bioviz.tokyo/functree/help).

FuncTree implements various node calculation options to achieve optimal functional reconstruction. User input data can be a list of KO composition or a list of relevant genes, as FuncTree will be able to calculate and reconstruct the functional profile for the entire functional hierarchy. User may also choose to conduct statistical testing between their input data and a pre-defined background data to calculate each node's enrichment, in order to create a tree map that shows the uniquely enriched functions of that omics data.

The customized option will be updated dynamically on to the tree map by default, and the result mapping may be exported for download in graphical format. Currently, FuncTree can export the tree map in SVG format, and we are expecting FuncTree to support several other graphical formats in future update. FuncTree also allows user to download the Expression table in plain text format.

### Data Mapping

**Fig 2:**
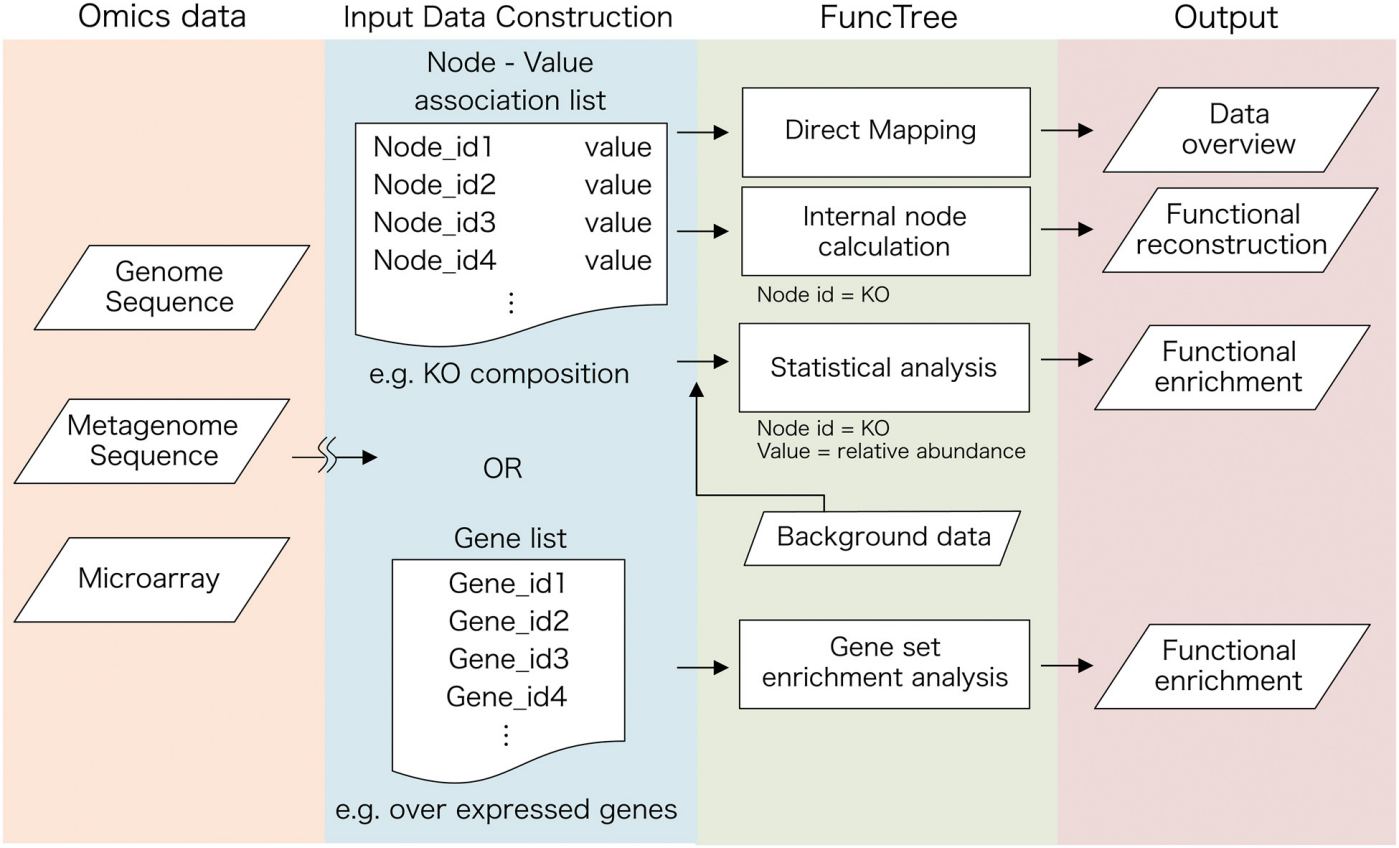
Flowchart for data mapping using FuncTree. FuncTree is a web-based application that is capable of analyzing and visualizing the functional characteristic of large-scale omics data. As an input, FuncTree expects either a list of node-value association, which associates each node with a designated value, or a list of genes. Value for each node would have to be calculated locally by user, and may represent a number of different variables such as relative abundance. Gene list may represent a list of over-expressed genes in a microarray experiment. Depending on the Input, FuncTree is able to perform various visualizations such as, (i) Direct mapping: directly map the value onto the designated node, (ii) Internal node calculation: Calculate the functional potential of the upper four layer from KO composition, (iii) Statistical analysis: Conduct enrichment analysis for each layer from KO composition against a predefined background data of several human metagenomic dataset, and (iv) Gene set enrichment analysis: Conduct enrichment analysis for each layer from gene list.

There are currently two types of input data format that FuncTree expects in order visualize the data (Fig 2). The first is the “node-value association list”, which is a list containing node id on the first row and a numerical value on the second row. Node id is the name or the identification number of biological functions within the Functional Tree, including KO, module, and Pathway. A full list of name and identification number of nodes assigned in the Functional Tree is available for download from FuncTree’s online data page (http://bioviz.tokyo/functree/data). The numerical value on the second row may represent various variables depending on the user’s intended visualization. It may represent the p-value of an enrichment analysis, which represents the statistical significance of that function, relative abundance of the function, or it can simply represent the direct size of the circle that will be mapped onto the node. For example, user may create an input data of a KO composition of metagenomic data, which is a list of KO and its relative abundance, using the following process.

1. Acquire short read DNA sequence using high throughput DNA sequencing technology.
2. Calculate the KO composition from the short read DNA sequence, using functional annotation server such as MG-RAST [4] or KAAS [5].

Depending on the input data type and the option selected, FuncTree would conduct three different types of mapping method.

1. *Direct mapping*: FuncTree will directly map the input value onto the designated node, and will draw a circle with the size corresponding to the input value. Optionally user may normalize the input value in order to fit it within a set range of pixel size to achieve optimal visualization. Additionally, the direct mapping allows user to assign additional attributes to the input data, including color and opacity.
2. *Internal Node Calculation*: If the input data is a list with KO number on the first row and a numerical value on the second row, FuncTree is able to calculate the value of the nodes in the upper layer (Module/Pathway) by computing the average or summation of the value of KOs assigned under it. For example, if the input data is a list of KO composition, FuncTree is able to calculate and reconstruct the functional composition of the remaining functional layer (Module/Pathway/Biological Process/Biological Category).
3. *Statistical Analysis (Enrichment analysis)*: If the input data is a list of KO composition, FuncTree is able to compute the statistical significance of the data, by comparing the data against FuncTree’s pre-defined background dataset. Currently FuncTree allows user to compare their metagenomic data against pre-defined metagenomic background data, which was sampled from different human body sites, acquired by the Human Microbiome Project [6].

The second type of input data format that FuncTree is able to process is a list of genes. For example this list can be a list of over-expressed genes of a particular cell acquired in a microarray experiment. FuncTree would conduct a gene set enrichment analysis to identify functions that are enriched in that list, by statistically comparing it against the genome of the organism, which that gene list originates from. Currently FuncTree expects the genes to be in KEGG gene id format, however we are expecting FuncTree to be able to accept other format such as COG/eggNOG, Uniprot, and NCBI gene ID in future update.

## Results and Discussion

To evaluate the validity of FuncTree's mapping and analysis capability, we provide three case stories to illustrate the use of FuncTree by mapping genomic, metagenomic, and transcriptomic data.

### Pan-genomic mapping: Functional enrichment of *Escherichia*, *Mycobacterium*, and *Streptomyces*

**Fig 3:**
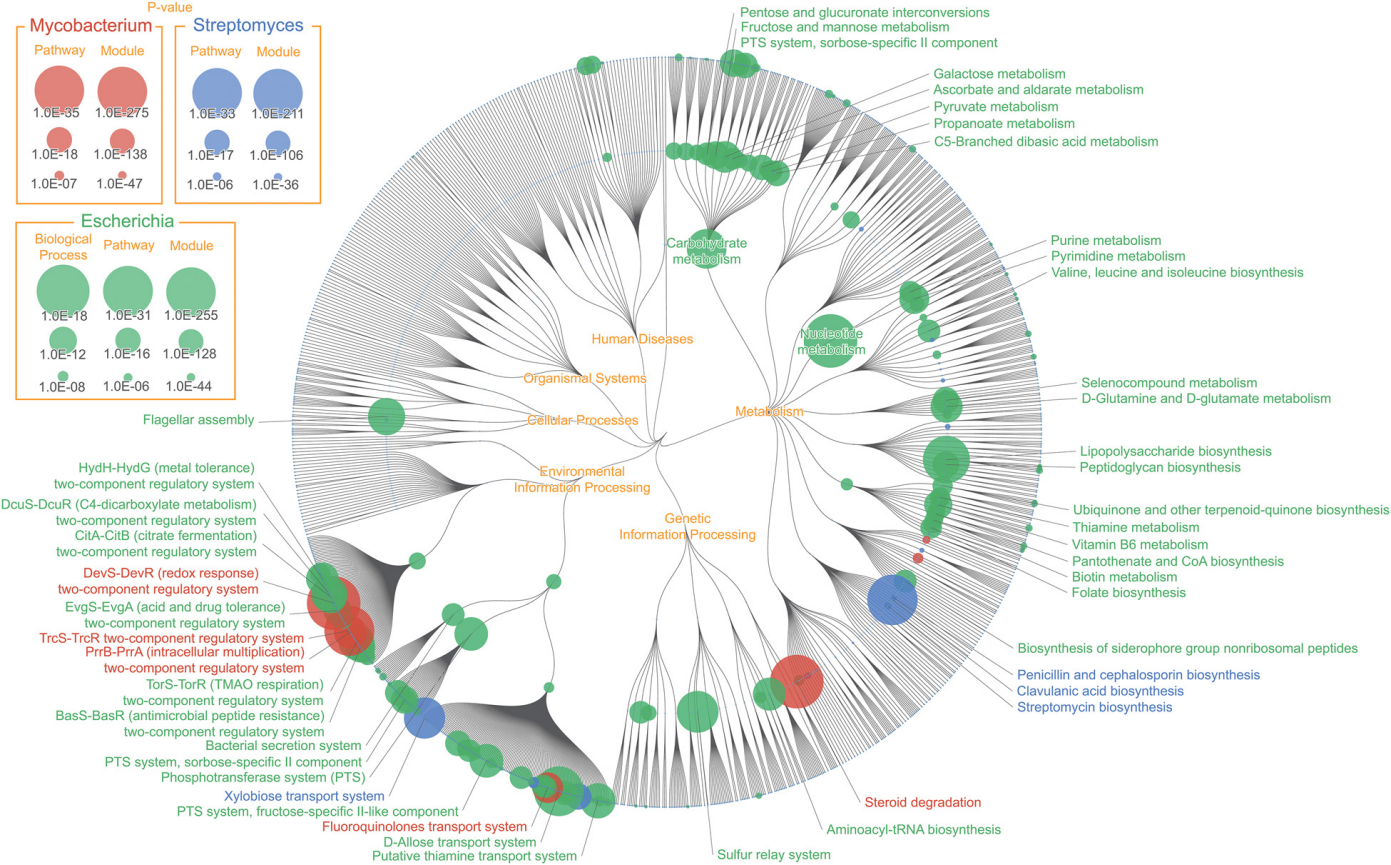
Pan-genomic mapping: Functional enrichment of *Escherichia*, *Mycobacterium*, and *Streptomyces*. Color node represents biological functions that were uniquely enriched in the pan-genomic data of *Escherichia* (green), *Mycobacterium* (red), and *Streptomyces* (blue). Node size corresponds with the inverse p-value calculated using Wilcoxon signed-rank test, representing how “uniquely” enriched that function was. “Lipopolysaccharide biosynthesis (map00540)”, “Flagellar assembly (map02040)”, and “D-Allose transport system (M00217)” were uniquely enriched for *Escherichia*, "Steroid degradation (map00984)", "DevS-DevR (redox response) two-component regulatory system (M00482)", and "TrcS-TrcR two-component regulatory system (M00463)" for *Mycobacterium*, "Clavulanic acid biosynthesis (map00331)", "Xylobiose transport system (M00619)", and "N-Acetylglucosamine transport system (M00205)" for *Streptomyces*.

The exponential growth of large-scale microbial genomic data has made it possible for biologists to explore organism's genetic information on a more comprehensive level. Analyzing pan-genome, which is the total list of genes present in the same taxon (e.g. species or genus) across different strains [7], has allowed biologists to explore the diverse variation of gene content among different strains of the same bacterial species. Pan-genomic analysis has provided evolutionary insights on various organisms including *Escherichia coli* [8] and *Haemophilus influenza* [9], and has also proven effective in identifying strain-specific virulence factor of pathogenic *Legionella pneumophila* [10]. Here we show FuncTree's application to pan-genomic analysis by conducting functional enrichment analysis of three different types of bacterial genera, and visualizing the biological functions that were uniquely enriched in each bacterium (Fig 3). Each genus’s pan-genomic data were statistically compared against the genomic data of all remaining prokaryotes to calculate enrichment (S1 Table).

*Mycobacterium*'s ability to survive within the host macrophage is one of the major reasons for it becoming a worldwide health problem [11]. Although the specific details and mechanism of how *Mycobacterium* survives within the host cell is yet unclear, recent study suggests that its ability to catabolize cholesterol in the host cell plays an important role in *Mycobacterium* survival and pathogenicity [12]. FuncTree was able to visually represent this by showing "Steroid degradation (map00984)" as the most uniquely enriched pathway of *Mycobacterium*. Additionally, a number of two-component signal transduction system such as, "DevS-DevR (redox response) two-component regulatory system (M00482)", a hypoxic responsive module which is crucial for *Mycobacterium* survival during dormancy, and "PrrB-PrrA (intracellular multiplication) two-component regulatory system (M00462)", which is associated with intracellular multiplication in murine macrophage, were uniquely enriched modules of *Mycobacterium*.

The average chromosome size of *Streptomyces* is approximately 8~9 Mbp long, making it one of the largest bacterial genome, and it is also known to be responsible for producing two-thirds of clinically useful antibiotics. The mapping result shows two pathways responsible for antibiotic biosynthesis, "Clavulanic acid biosynthesis (map00331)" and "Penicillin and cephalosporin biosynthesis (map00311)" being the uniquely enriched functions of *Streptomyces*. Clavulnic acid is a β-Lactamase inhibitor that is often times used in combination with other penicillin group antibiotics. *Streptomyces clavuligerus* is known to biosynthesize Clavulnic acid from glyceraldehyde 3-phosphate and arginine [13].

Pan-genomic analysis is an integral approach to understand the general characteristic of bacterial species, and to discover new evolutionary and functional insights. Here we have shown an example that unifies multiple genomic data into pan-genomic data, and use FuncTree to visualize the general functional feature of three distinct types of bacterial genera in to a single overview map. We believe FuncTree’s visualization capability to provide a holistic functional overview will prove extremely useful in future pan-genomic analysis, such as identifying functional differences between pathogenic and non-pathogenic strains.

### Metagenomic mapping: Functional variability and enrichment of human gut microbiome

**Fig 4:**
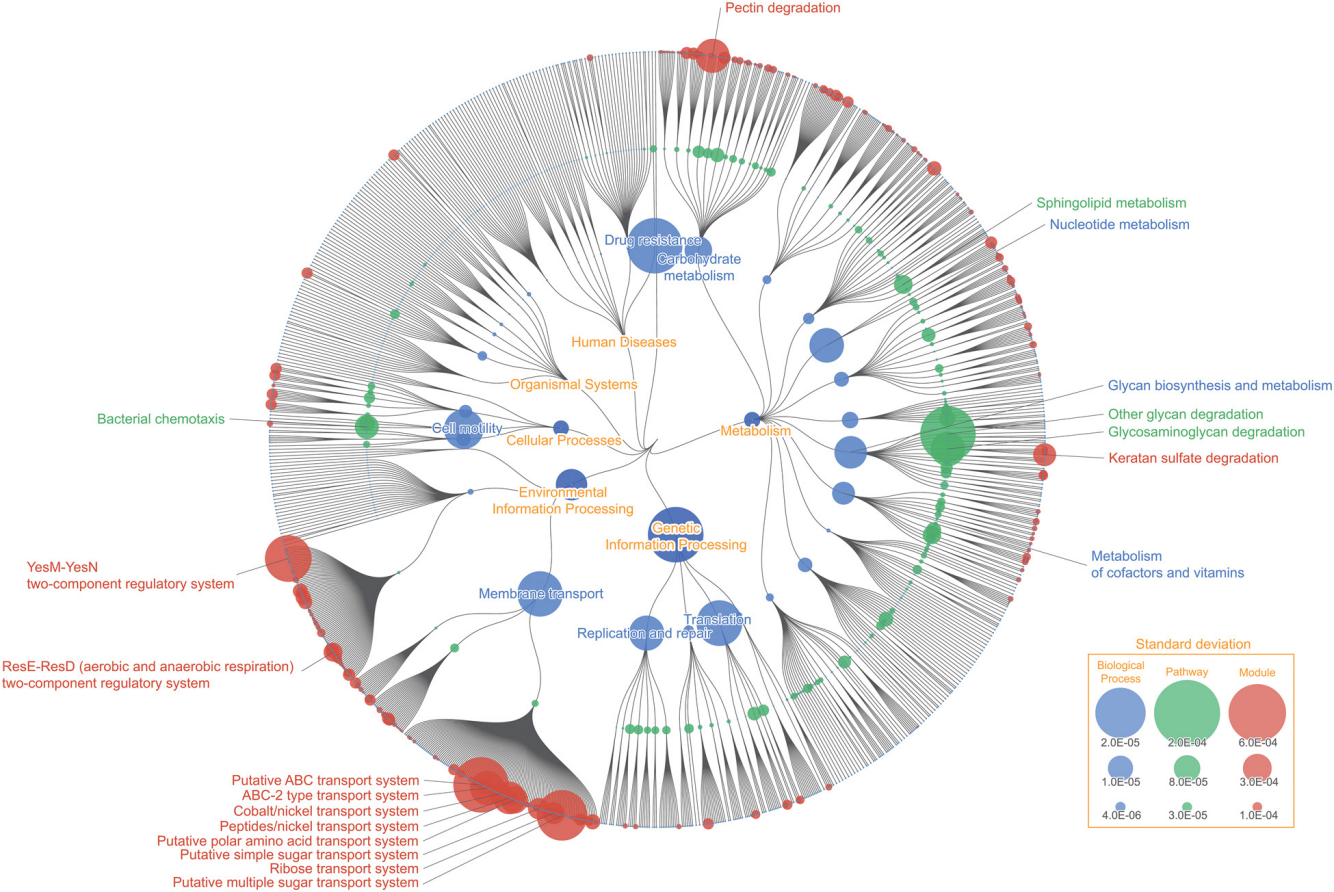
Functional variability of human gut microbiota. Color node represents biological functions that were highly variant among fecal samples from different individuals. Node size corresponds to the value of standard deviation of the KO’s relative abundance assigned to that function. “Other glycan degradation (map00511)”, “Glycosaminoglycan degradation (map00531)”, and “Bacterial chemotaxis (map02030)” showed high variability among the KEGG Pathway layer, while “Pectin degradation (M00081)”, "Peptides/nickel transport system (M00239)", "Ribose transport system (M00212)", and "ABC-2 type transport system (M00254)" showed high variability among the KEGG Module layer. The difference in variability pattern between the KEGG Pathway layer and KEGG Module layer in this mapping illustrate the potential problem of focusing on a particular functional category, and shows the necessity for overviewing the broad functional potential across different functional layers.

**Fig 5:**
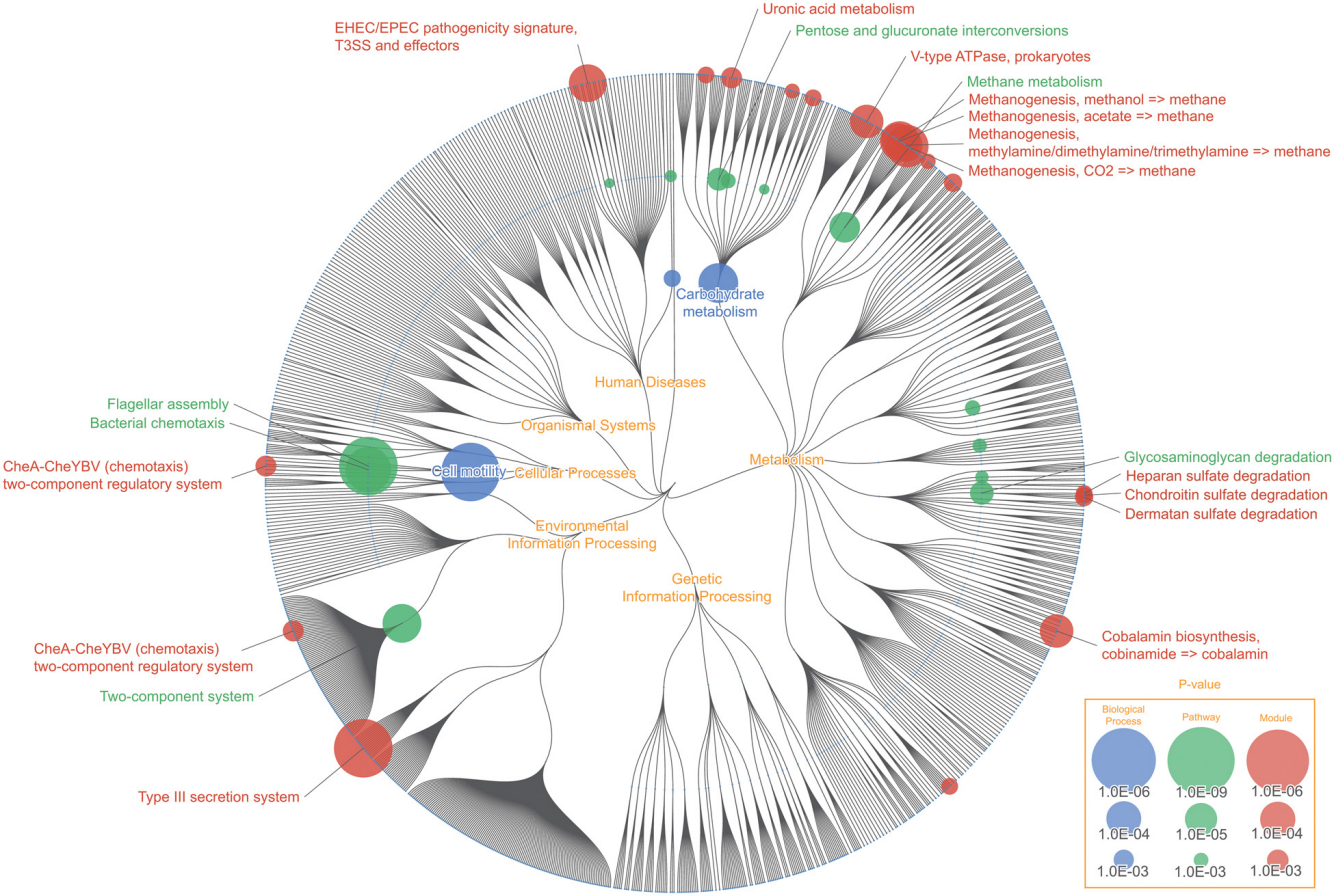
Functional enrichment of human gut microbiota. Color node represents biological functions of the human gut microbiota that were uniquely enriched compared to microbiota of other human body sites. For the KO functional layer, node size corresponds to the inverse p-value of Wilcoxon signed-rank test, and for the remaining functional layers, node size corresponds to the inverse p-value of Fisher’s exact test. Pathways that were uniquely enriched in the human gut microbiota were, “Flagellar assembly (map02040)”, “Bacterial chemotaxis (map02030)”, “Methane metabolism (map00680)”, and “Glycosaminoglycan degradation (map00531)”. Modules including “Type III secretion system (map03070)” and “Cobalamin biosynthesis, cobinamide = > cobalamin (M00122)” were shown to be highly enriched on the KEGG Module layer, but not so on the KEGG Pathway layer, which exemplifies a situation where the enrichment pattern differs based on different functional layer, thus supporting the importance for a broad visual overview of the data.

The human intestinal tract is one of the most intensively studied environments in the field of metagenomic, for its microbial and functional composition is suggested to have significant impact on human health and diseases. Here we show FuncTree's application to metagenomic data by analyzing functions that were variant among fecal samples from different individuals (Fig 4), and conducting functional enrichment analysis to identify biological functions that were uniquely enriched in the intestinal environment (Fig 5).

### Functional variability of human gut microbiota

Node size represents the standard deviation of each KO’s relative abundance assigned to each biological function, across 1,267 samples of gut metagenome (Fig 3). "Other glycan degradation (map00511)" which is responsible for degrading N-glycan and Ganglioside was highly variant among different samples (S2 Table). *Bifidobacterium* is known to degrade and remove N-glycan from diary products such as milk [14], while Ganglioside is known to inhibit bacterial toxin and virus from binding with intestinal epithelium [15]. Variability in this pathway may be representing the variability among human's ability to digest dairy products, and difference in immunity against pathogenic bacteria and viruses. Module responsible for "Pectin degradation (M00081)" was also highly variant among samples. Pectin is a heteropolysaccharide that are often times found in cell walls of plants. 70 ~ 90% of Pectin are degraded into butyric acid and short-chained fatty acid by gut microbes to be utilized as energy source of the host species. Variability of this module could represent the difference among individuals to digest and utilize dietary fibers. Many of the "ABC transporter" modules including, "Peptides/nickel transport system (M00239)", "Ribose transport system (M00212)", and "ABC-2 type transport system (M00254)" were also highly variant among samples. ATP transporters are membrane transport protein, which utilize ATP energy to translocate various substances across intra- and extracellular membrane, and are responsible for carrying out various cellular processes. Variability in these functions could be representing the broad difference of biological processes, such as difference in ability to digest certain substance as nutrients, and the difference in biological response against pathogens and drugs, among different gut microbiome. It should be noted that the variability pattern differs between Pathways and Modules. This may be due to the limitation of KEGG's manual categorization, where some pathway contains no defined modules (e.g. map00511), whereas other pathways are not categorized enough and contains too many modules (e.g. map02010). This leads to a situation where concentrating on a single functional layer can lead to loss of information or misinformation. When you concentrate on the module layer, it shows that functions categorized under "ABC transporters" and "Two component system" are the most variant functions. However this seems only so because certain functions such as "Other glycan degradation (map00511)" and "D-Alanine metabolism (map00473)" do not have any defined modules categorized under it, and there is no module to represent these functions. As a matter of fact, when you concentrate on the pathway layer, you can see that "ABC Transporters" and "Two component system" are relatively less variant. FuncTree's ability to visualize the result across multiple functional layers thus provides a more accurate and holistic understanding of the data, compared to conventional visualization method, where you only concentrate on a single functional layer.

### Functional enrichment of human gut microbiota

The color nodes represent functions that were uniquely enriched in gut microbiome (Fig 4). Metagenomic data of human intestine were statistically compared against metagenomic data of other human body sites (e.g. oral, nasal, skin, urogenital). For the KO functional layer, node size represents inverse p-value of Wilcoxon signed-rank test, and for the remaining functional layers, node size represents inverse p-value of Fisher’s exact test.

The result visualization shows that pathway for "Methane metabolism (map00680)" was among the most uniquely enriched functions of the human gut microbiota (S2 Table). In particular modules responsible for methanogenesis (M00356, M00357, M00563, M00567) were highly enriched. It is suggested that methanogenic archaea, which is responsible for synthesizing methane, plays an important role in the gut ecosystem, by metabolizing hydrogen, which are produced as a result of fermentation of carbohydrate, and oxidize them into methane. In the human gut, *Methanobrevibacter smithii* is known to be the predominant archaeon responsible for oxidizing hydrogen, produced during the digestion of polysaccharides, thus increasing the efficiency of energy and nutrient uptake [16]. Recent study also suggests that methane produced by intestinal methanogen may have an influence on the pathogenesis of constipation, irritable bowel syndrome, and obesity [17]. Although certain species of *Methanobrevibacter*, such as *M*. *oralis*, were isolated from non-gut human environment [18], *Methanobrevibacter* such as *M*. *smithii* and *M*. *ruminantium* are often times isolated from the human gut. This could explain why "Methane metabolism" was identified as a unique function of the gut environment.

"Glycosaminoglycan degradation (map00531)" was also identified as one of the uniquely enriched function of the gut environment, with all three modules assigned under it, also being highly enriched. This result was consistent with the result of metabolic reconstruction of the gut metagenome, using the HUMAnN pipeline [19]. Glycosaminoglycan (GAG) are mucopolysaccharides that are often found in connective tissues of animals. The three modules that were enriched were, "Heparan sulfate degradation (M00078)", "Chondroitin sulfate degradation (M00077)", and "Dermatan sulfate degradation (M00076)". All of three modules were involved with the degradation of animal proteoglycan for carbohydrate utilization for microbes. Past study has also reported that glycosaminoglycan degradation was identified as enriched function among the *Bacteroides* species [20], which are one of the predominant bacterial genera of the gut microbiome.

Numerous studies have shown strong association between the human gut microbiota and various human diseases. It is expected that the number of gut metagenomic data will keep increasing exponentially, and a strong platform for functional analysis of metagenomic data is becoming increasingly important. We believe that FuncTree would become an indispensible part of the initial phase of future comparative metagenomic analysis, by providing holistic overview of functional similarities and differences between multiple metagenomic datasets.

### Transcriptomic mapping: Functional enrichment mapping, reconstructed from mRNA expression data of two types of human cell

**Fig 6:**
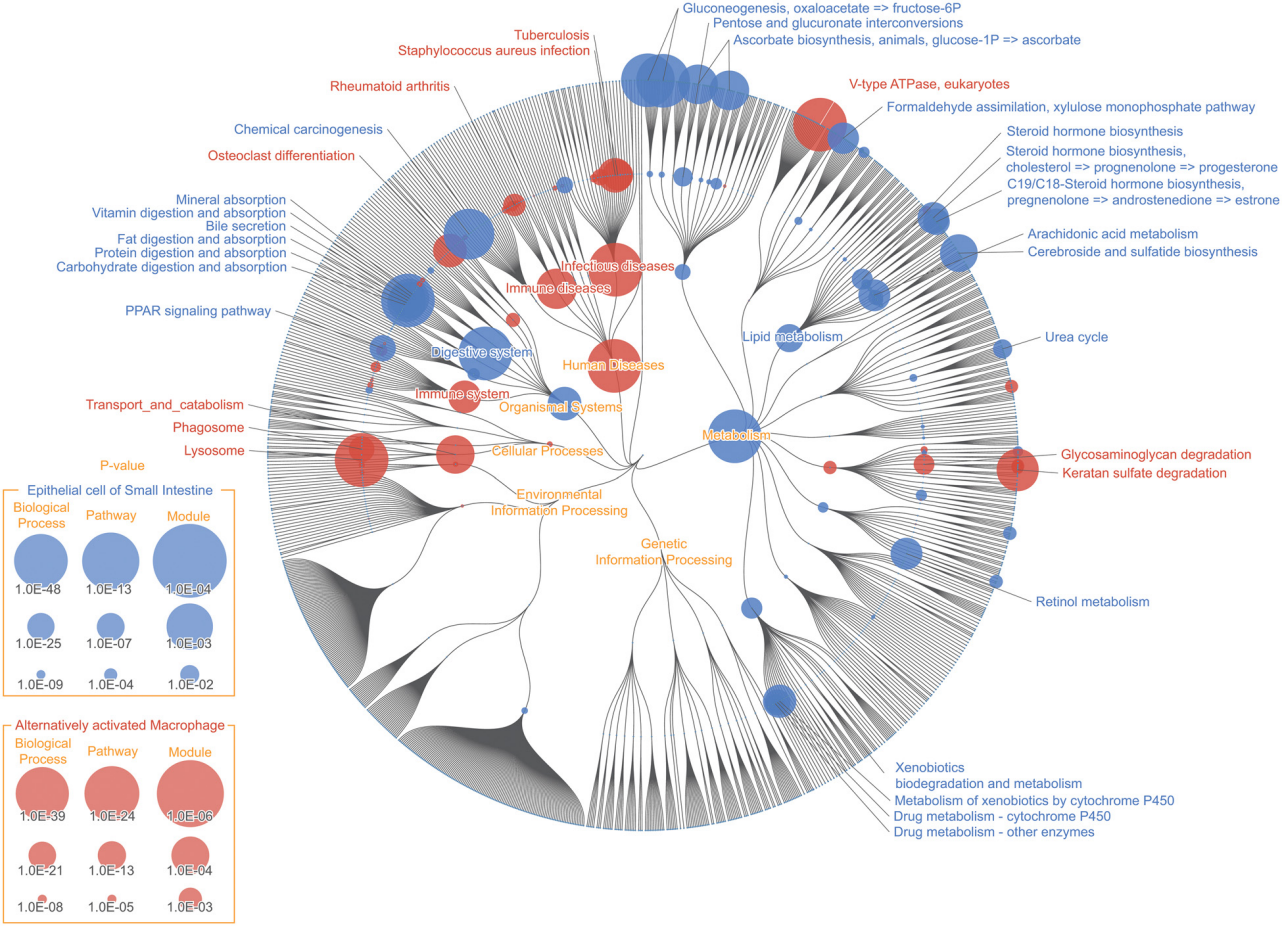
Transcriptomic mapping: Epithelial cell of small intestine and alternatively activated macrophage. Color node represents biological functions that were enriched in the “Epithelial cell of small intestine”(Blue) and “Alternatively activated Macrophage”(Red). Node size corresponds to inverse p-value of enrichment analysis, based on Fisher’s exact test, of the 590 over-expressed genes for “Epithelial cell of small intestine” and 309 over-expressed genes for “Alternatively activated Macrophage”, detected in microarray experiment. Digestive functions including "Fat digestion and absorption (map04975)", "Vitamin digestion and absorption (map04977)", "Mineral absorption (map04978)", "Bile secretion (map04976)", and "Pancreatic secretion (map04972)" were uniquely enriched, validating our knowledge about intestinal epithelium. Pathways under the category “Transports and catabolism” including "Lysosome (map04142)" and "Phagosome (map04145)" were uniquely enriched in “Alternatively activated Macrophage”, Pathways categorized under "Digestive system", including "Fat digestion and absorption (map04975)", "Vitamin digestion and absorption (map04977)", "Mineral absorption (map04978)", "Bile secretion (map04976)", and "Pancreatic secretion (map04972)" were significantly enriched in "Epithelial cell of small intestine", which validates our knowledge that intestinal epithelium is where many of the digestive process takes place (S3 Table). Another characteristic of this cell was the enrichment of pathways categorized under "Endocrine system" including, "PPAR signaling pathway (map03320)", "Leukocyte transendothelial migration (map04670)", "Renin-angiotensin system (map04614)", "Insulin secretion (map04911)", "Ovarian steroidogenesis (map04913)", "Melanogenesis (map04916)", and "Thyroid hormone synthesis (map04918)". This also is consistent with known knowledge, that the epithelium of the small intestine is also comprised of enteroendocrine cell which produces gastrointestinal hormones to initiate digestive response and protective function against harmful substances [21]. Functions that characterize the “Alternatively activated macrophage” were pathways categorized under "Transports and catabolism" which includes, "Lysosome (map04142)", "Phagosome (map04145)", "Cell adhesion molecules (map04514)", and "Cytokine-cytokine receptor interaction (map04060)". “Lysosome (map04142)” and “Phagosome (map04145)” plays a crucial role during the process where macrophage ingests target pathogens.

Advancement of sequencing technology has allowed biologists to comprehensively explore the entire RNA transcripts expressed in a specific cell under specific conditions. Here we show FuncTree's application to transcriptomic data, by mapping mRNA expression data from "epithelial cell of small intestine" and "alternatively activated macrophage", to visualize biological functions which were enriched in each cell (Fig 6).

Analyzing gene expression patterns is essential in comprehending complex functions of biological mechanism, which would further contribute to our understanding of diseases such as cancers and diabetes. Here we have shown FuncTree’s application to transcriptomic analysis by mapping two different types of human cell expression data. We believe that FuncTree’s flexibility to map various types of omics data would make it an integral platform for future large-scale omics analysis.

### Comparison with other tools

In order to quantify the unique key features of FuncTree, we have compared FuncTree’s calculation and visualization capability against three other visualization applications including, iPath v2.0 [1], KEGG Atlas [22], and PathwayProjector [2], using several functional categories including, (i) User interface, (ii) Visual customization, (iii) Functional calculation, (iv) Input data type, (v) Data output, and (vi) Functional visualization (Table 1). All tools provides integrated pathway map with pan/zoom functionality and basic mapping capability. FuncTree instead provides a more holistic map of the entire functional hierarchy, which not only incorporates the information of each pathway, but also the information of each module and ortholog, and further provides information about the hierarchical relationship of each function. Additionally, FuncTree provides powerful functional calculation methods to reconstruct the functional profile of a given dataset, or to conduct statistical enrichment analysis against a different background data. We believe that this integration of visual and analytical capability into a uniformed web application is what makes FuncTree unique from other similar web services.

**Table 1:** Comparison of FuncTree’s key features with other visualization tools.

Function category	Functions subcategory	Web server			
		Functree	iPath 2.0	KEGG Atlas	PathwayProjector
**User Interface**	zoom/pan	o	o	o	o
	mouse over popup	o	o	o	o
**Visual customization**	Size	o	o		o
	Color	o	o	o	o
	opacity	o	o		
**Functional calculation**	Functional reconstruction	o			
	Statiscal enrichment	o			
**Input data type**	KEGG orthology	o	o	o	o
	KEGG pathway	o		o	o
	KEGG module	o		o	o
	KEGG gene	o			
	COG/eggNOG		o		
	UniProt		o		
	NCBI gene ID		o		
	Multi-point data	-			o
**Data output**	SVG	o	o	o	o
	Table	o			
**Functional visualization**	Hierarchical Overview	o			
	Pathway	o	o	o	o
	Module	o	o	o	
	KO	o	o	o	

Taken together, the three mapping application and the comparative uniqueness of FuncTree strongly confirms the ability of FuncTree for functional visualization. We believe FuncTree’s ability to map various types of large-scale omics data will make it an indispensible visualization tool for biologists.

## Method

### Construction of Functional Tree

The Functional Tree: a circular dendrogram mapping background, which visualize the hierarchical classification of known biological functions, was created using data from KEGG Brite Database ver. 2013-9-27 [3]. Since the specific hierarchical relationship of biological functions between different functional layer were mostly undefined, we have first parsed through all of the 28,505 KEGG Orthology and manually defined the hierarchical relationship between them. As a result, 20,687 KOs were hierarchically assigned to 676 modules and 365 pathways. The remaining 7,818 KOs that could not be hierarchically assigned to any upper functional layer, were assigned under "Undefined Biological Category". The resulting hierarchically classified biological functions were output in JSON format. Finally, the JSON data was visualized into a circular dendrogram by using D3.js, a javascript library for data visualization. Data mapping and statistical calculation was implemented using Javascript and CGI application.

### Pan-genomic mapping: Functional enrichment of *Escherichia*, *Mycobacterium*, and *Streptomyces*

**Fig 7:**
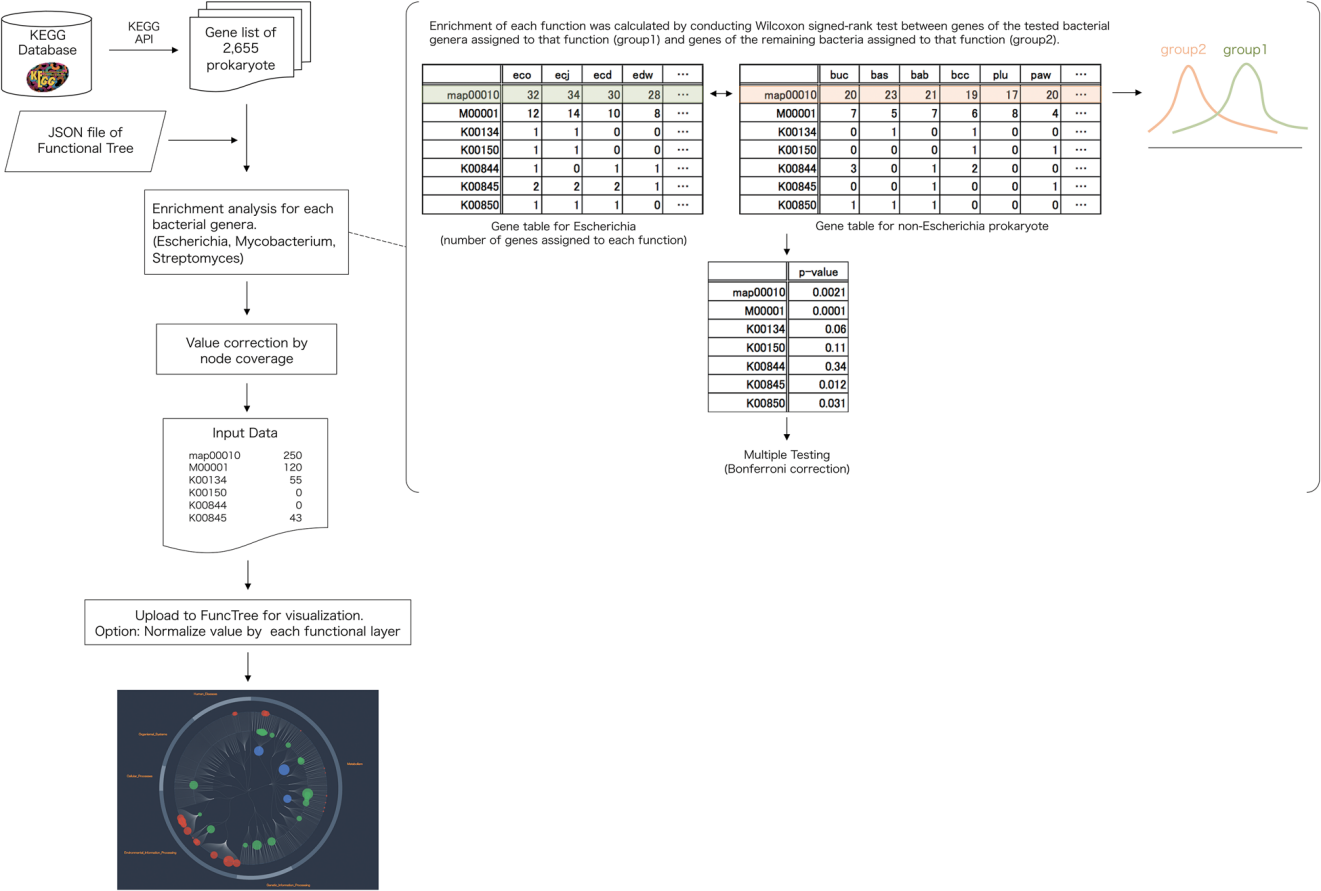
Flowchart for pan-genomic mapping. Gene list of 2,655 prokaryotic organisms were downloaded from the KEGG database (http://www.genome.jp/kegg/). Using the hierarchical structure of the “Functional tree”, each nodes enrichment were calculated by conducting Wilcoxon signed-rank test between, genes of the tested bacterial genera (*Escherichia*, *Mycobacterium*, *Streptomyces*) that was assigned to that node, and the genes of the remaining prokaryotes assigned to that node. Each node’s value was corrected using node coverage to produce the input data. Final visualization and normalization was conducted using FuncTree.

Original pan-genomic data for each bacterium were downloaded using REST-style KEGG API (Fig 7). Using the KEGG API, gene list of all 2,655 bacteria were downloaded. For each node (biological function) in the Functional Tree, Wilcoxon signed-rank test was used to calculate the difference in distribution of gene numbers of the tested bacterial genera (*Escherichia*, *Mycobacterium*, *Streptomyces*) that was assigned to that function, and the gene number of the remaining bacteria that was assigned to that function. The first group was an array of gene number that is assigned to the tested bacterial genera (*Escherichia*, *Mycobacterium*, *Streptomyces*) and is associated with the biological function. The second group was an array of gene number that is assigned to remaining bacteria (gene list for all prokaryote minus the gene list of the tested bacterial genera) and is associated with the biological function. The two groups were statistically compared using Wilcoxon signed-rank test, with Bonferroni correction.

In order to adjust the inequality between functions, depending on the number of KO assigned under it, coverage for node *j* (function) was calculated using the following calculation.

$$cov_{j,k}=\frac{KO_{exp,\;j,k}}{KO_{total,\;\;j}}$$ (1)

Where *KO*_*total*,*j*_ is the total number of KO assigned under node *j*, and *KO*_*exp*,*j*,*k*_ is the number of KO that is associated with the tested bacterial genera: bacteria *k*. A KO was considered to be associated with the bacteria *k* if even one gene of bacteria_k_ was assigned to that KO. If the null hypothesis was dismissed (p < 0.05) after the statistical analysis, value for node *j* (function) was calculated using the following equation.

$$val_{j,k}=\;log\frac{1}{P_{wilcox,j,k}}\times \;cov_{j,k}$$ (2)

Where *P*_*wilcox*,*j*,*k*_ is the resulting p-value of the Wilcoxon signed-rank test with Bonferroni correction, computing the statistical significance of gene number assigned to bacteria_k_, that was associated as part of node *j*. The list of node name and value was uploaded to FuncTree to finalize visualization.

### Metagenomic mapping: Functional variability of human gut microbiota

KO composition of 1,267 human gut metagenome samples [23], were used to visualize the functional variability of gut microbiota. For each node (biological function) in the Functional Tree, the standard deviation of relative abundances of KOs assigned to that function across 1,267 samples was calculated. In order to adjust the inequality between functions, depending on the number of KO assigned under it, node coverage was calculated using the following equation.

$$cov_{j,human\_gut}=\frac{KO_{exp,\;j,human\_gut}}{KO_{total,\;\;j}}$$ (3)

Where *KO*_*total*,*j*_ is the total number of KO assigned under node *j*, and *KO*_*exp*,*j*,*human_gut*_ is the number of KO that is associated with the human gut. A KO was considered to be associated with the human gut if even one sample of the human gut metagenome was assigned to that KO. Value for node *j* (biological function) was calculated using the following equation.

$$val_{j,human\_gut}=\;\sigma_{j}\;\times \;cov_{j,human\_gut}$$ (4)

Where σ_*j*_ is the standard deviation of relative abundances of KOs assigned to node *j*, and σ_*j*_ is the coverage value for node *j*. The list of node name and value was uploaded to FuncTree to finalize visualization.

### Metagenomic mapping: Functional enrichment of human gut microbiota

**Fig 8:**
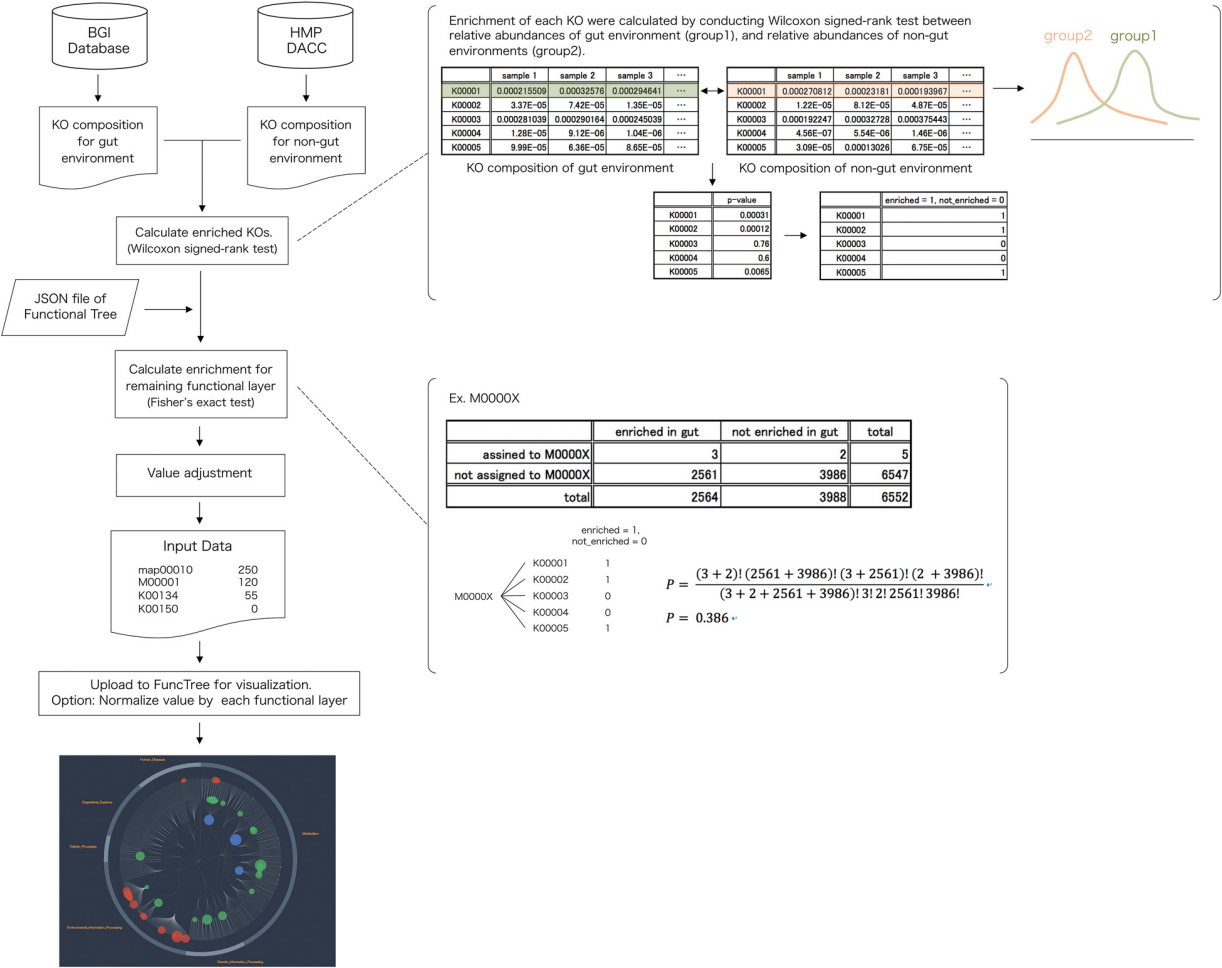
Flowchart for human gut metagenomic mapping. KO composition for gut environment and non-gut environment were downloaded from the BGI database (http://meta.genomics.cn) and HMP DACC (http://www.hmpdacc.org/). Calculation of enriched KO was conducted using Wilcoxon signed-rank test between relative abundance of the gut environment and the relative abundance of non-gut environment. Using the hierarchical structure of the “Functional tree”, calculation for the enrichment of the remaining four layers was conducted using Fisher’s exact test. Final visualization and normalization was conducted using FuncTree.

KO composition of 1,267 human gut metagenome samples were used to visualize the functional enrichment of gut microbiota (Fig 8). As for the original data, we have used the KO composition constructed from the integrated gene catalogue of the human gut metagenome, which comprise of 9,879,896 genes [23]. In order to identify the enriched functions of the human gut microbiota, this data was statistically compared against KO composition of other non-gut human metagenome (oral, nasal, skin, urogenital) acquired from HMP DACC [6]. For each node (biological function) in the Functional Tree, statistical calculation was conducted to evaluate whether the human gut metagenome's collective relative abundance of the questioned function was significantly larger than the non-gut metagenome's collective relative abundance of the questioned function. For the KO functional layer, Wilcoxon signed-rank test was used to calculate the difference in distribution between the relative abundance of the gut and non-gut body sites, to identify KOs that were enriched. For the remaining functional layers, Fisher’s exact test was used to calculate the statistical significance of each function using the following equation.

$$P_{fisher,j}=\;\frac{\left(\sum KO_{j,\;e}\;+\;\sum KO_{j,\;\bar{e}}\right)!\left(\sum KO_{\bar{j},\;e}\;+\;\sum KO_{\bar{j},\;\bar{e}}\right)!\left(\sum KO_{j,\;e}\;+\;\sum KO_{\bar{j},\;e}\right)!\left(\sum KO_{j,\;\bar{e}}\;+\;\sum KO_{\bar{j},\;\bar{e}}\right)!}{\left(\sum KO_{j,\;e}\;+\;\sum KO_{j,\;\;\bar{e}}+\sum KO_{\bar{j},\;e}\;+\;\sum KO_{\bar{j},\;\bar{e}}\right)!\sum KO_{j,\;e}!\sum KO_{j,\;\bar{e}}!\sum KO_{\bar{j},\;e}!\sum KO_{\bar{j},\;\bar{e}}!}.$$ (5)

Where *P*_*fisher*,*j*_ is the resulting p-value for node *j* (function), Σ*KO*_*j*,*e*_ is the total number of enriched KO that were assigned to node *j*, $\sum {KO}_{j,\;\bar{e}}$ is the total number of non-enriched KO that were assigned to node *j*, $\sum {KO}_{\bar{j},\;e}$ s the total number of enriched KO that were not assigned to node *j*, $\sum {KO}_{\bar{j},\;\bar{e}}$ is the total number of non-enriched KO that were not assigned to node *j*. Finally, the input value for node *j* was calculated using the following equation.

$$val_{j}=\;log\frac{1}{P_{fisher/wilcox}}$$ (6)

Where *P*_*fisher/wilcox*_ is the resulting p-value for Wilcoxon signed-rank test, for the KO functional layer, and Fisher’s exact test for the remaining functional layers. The list of node name and value was uploaded to FuncTree to finalize visualization.

### Functional enrichment mapping, reconstructed from mRNA expression data of two different types of human cell

Raw expression data of two distinct cell type: "alternatively activated macrophage" and "epithelial cell of small intestine" was acquired from GEO microarray repository [24]. Probe data of the two cell types were statistically compared with that from other cell expression data in order to construct a list of genes that was over-expressed in each cell type. We have identified 309 over-expressed genes in "alternatively activated macrophage" and 590 over-expressed genes in "epithelial cell of small intestine" (S4 Table). In order to conduct functional enrichment analysis, the entire list of genes assigned to *Homo sapiens* was also downloaded from the KEGG database. For each node (biological function) in the Functional Tree, statistical calculation was conducted to evaluate whether the number of genes, which was expressed in a particular cell type, and was associated as part of the questioned biological function, was significantly larger than the number of genes, which is coded in the human genome, and was associated as part of the questioned biological function. Fisher's exact test with Bonferroni correction was used to calculate the statistical significance of each biological function.

$$P_{fisher,j,k}=\frac{(\sum g_{j,k}\;+\;\sum g_{j,\overset{-}{k}})!(\sum g_{j,k}\;+\;\sum g_{\overset{-}{j},k})!(\sum g_{j,\overset{-}{k}}\;+\;\sum g_{\overset{-}{j},\overset{-}{k}})!(\sum g_{\overset{-}{j},k}\;+\;\sum g_{\overset{-}{j},\overset{-}{k}})!}{(\sum g_{j,k}\;+\;\sum g_{j,\overset{-}{k}}+\;\sum g_{\overset{-}{j},k}\;+\;\sum g_{\overset{-}{j},\overset{-}{k}})!\sum g_{j,k}!\sum g_{j,\overset{-}{k}}!\sum g_{\overset{-}{j},k}!\sum g_{\overset{-}{j},\overset{-}{k}}!}$$ (7)

Where Σ*g*_*j*,*k*_ is the total number of genes expressed in cell-type *k* that was associated with node *j* (function), $\sum g_{j,\bar{k}}$ is the total number of genes coded in the human genome but not expressed in cell-type_k_ that was associated with node *j*, $\sum g_{\bar{j},\;k}$ is the total number of genes expressed in cell-type *k* but was not associated with node_j_, and $\sum g_{\bar{j},\bar{k}}$ is the total number of genes coded in the human genome but not expressed in cell-type *k* that was not associated with node_j_.

In order to adjust the inequality between functions, depending on the number of KO assigned under it, node coverage was calculated using similar methods as the pan-genomic mapping. If the null hypothesis was dismissed (p < 0.05) after the statistical analysis, value for node *j* and cell-type *k* was calculated using the following equation.

$$val_{j,k}=\;log\frac{1}{P_{fisher,j,k}}\times \;cov_{j,k}$$ (8)

Where *cov*_*j*,*k*_ is the coverage value for node *j* with cell-type *k*_,_ and *P*_*wilcox*,*j*,*k*_ is the resulting p-value of the Wilcoxon signed-rank test with Bonferroni correction, computing the statistical significance of gene number, which was expressed in cell-type *k*, and was associated as part of node *j*. The list of node name and value was uploaded to FuncTree to finalize visualization.

## Conclusion

The dramatic increase in quantity and complexity of high-throughput data is increasing the demand for a visualization method that uncovers key features of large-scale omics information. Here we provide FuncTree, a functional analysis and visualization web-application, which incorporates a novel visualization method and statistical calculation capability in order to analyze and visualize the functional potential of diverse types of omics information.

As the main feature of FuncTree, we have implemented a novel visualization method by constructing the Functional Tree map, a circular dendrogram representing the hierarchical relationship of biological functions defined in the KEGG database. This hierarchical visualization of biological functions provides two main advantages over conventional visualization methods. Firstly, users are able to overview the functional potential of the omics data, across multiple different functional layers, such as pathway, module, and KO. This allows user to view different functional patterns across different functional layers, which allows them to accurately strategize their approach for further detailed analysis of the omics data. Secondly, users are able to accurately identify the expression of overlapping functions, such as module and KO that are assigned to multiple different pathways.

We further validated FuncTree’s analysis and visualization capability by mapping pan-genomic, metagenomic, and transcriptomic data respectively. All three mapping showed that FuncTree was able to facilitate discovery by rearranging the omics data, strongly confirming FuncTree’s capability for functional analysis and visualization. Furthermore, mapping the functional variability of the human gut metagenome using FuncTree has identified that functional patterns of a given data may differ depending on different functional layer, thus confirming our point that a broad overview of all of the functional layer is necessary for a comprehensive understanding of the data. The metagenomic mapping of the human gut using FuncTree, was able to identify unique key functions associated with the gut environment.

The integration of the analytical capability to conduct functional reconstruction and enrichment, and the visual capability to visualize the broad functional potential of a data, will make FuncTree an integral tool for various kinds of omics base research. We are planning the update FuncTree’s visualization capability to allow it to visualize multi-point data, to accommodate for things such as time-series RNA-seq analysis. We also plan to develop an API, which will allow programmable access to FuncTree by end user and other applications.

## Supporting Information

S1 Table Pan-genomic mapping: Uniquely enriched functions of *Escherichia*, *Mycobacterium*, and *Streptomyces*. (XLSX)

S2 Table Human gut metagenomic mapping: Variant and enriched function of the human gut. (XLSX)

S3 Table Human cell transcriptomic mapping: Uniquely enriched functions of “Alternatively-activated macrophage” and “Epithelial cell of Small Intestine”. (XLSX)

S4 Table Over-expressed genes of “Alternatively-activated macrophage” and “Epithelial cell of Small Intestine”. (XLSX)
